# A qualitative study exploring the views of healthcare professionals regarding patients who have difficulty swallowing medicines

**DOI:** 10.1007/s11096-026-02129-9

**Published:** 2026-04-01

**Authors:** A. Harnett, C. Murphy, L. J. Sahm, S. Byrne, D. Lyons, M. O’Driscoll

**Affiliations:** 1 Pharmaceutical Care Research Group, School of Pharmacy, University College Cork, Cork, Ireland; 2 University Hospital Limerick, Dooradoyle, Limerick, Ireland; 3 Pharmacy Department, Mercy University Hospital, Grenville Place, Cork, Ireland

**Keywords:** Dysphagia, Hospital setting, Interprofessional, Qualitative, Solid oral dosage forms, Swallowing difficulty

## Abstract

**Background:**

In the hospital setting healthcare professionals (HCPs) are involved in medicines management for patients with difficulty swallowing solid oral dose forms (SODF), which can present unique challenges.

**Aim:**

To investigate HCPs’ views and identify resulting appropriate behaviour change strategies for HCPs to optimise the care of patients with difficulty swallowing SODF in an acute hospital setting.

**Method:**

Between 3rd June and 12th July 2024 qualitative, semi-structured, in-person/online interviews with HCPs working in an Irish hospital, were conducted. Initial open coding of transcripts generated non-hierarchical codes with subsequent categorisation into themes. The Theoretical Domains Framework (TDF) provided predetermined codes and facilitated a directed content analysis. Dominant TDF domains were identified and mapped to the Capability Opportunity Motivation-Behaviour (COM-B) model and Behavioural Change Wheel.

**Results:**

A total of 13 HCPs participated in the study (n = 12 female; n = 4 doctors, n = 3 nurses, and n = 2 dietitians, speech & language therapists and pharmacists respectively), with average interview length of 22 min (range 12–32 min). Eight TDF domains were found to be dominant: Memory Attention & Decision Processes; Environment, Context and Resources; Knowledge; Social/Professional Role and Identity; Goals; Beliefs about Consequences; Behavioural Regulation and Intentions. HCPs identified swallow assessments for medicines, decision support tools and multidisciplinary team (MDT) input as important enablers of safe patient care.

**Conclusion:**

HCPs require the provision of education, and training, suitable infrastructure and access to evidence-based information to appropriately care for patients with difficulty swallowing SODF in the acute hospital setting. Future work should seek to address these issues through tailored interventions.

**Supplementary Information:**

The online version contains supplementary material available at 10.1007/s11096-026-02129-9.

## Impact statements

- Early identification of patients with swallowing difficulties at admission, using a tick-box or questionnaire, or early swallow assessment, is crucial for patient care.
- Healthcare professionals reported needing education and training to address knowledge gaps, in relation to appropriate formulations and SODF modification.
- Interprofessional collaboration was valued as a support to good decision making, specifically pharmacists were identified as having the appropriate expertise on medicines modification and alternative formulations.

## Introduction

Swallowing is a critical and complex physiological process [1]. Impairment of swallow, or dysphagia, affects a patient’s ability to swallow medicines [2], in particular solid oral dose forms (SODF), such as tablets and capsules [3]. Patients can become dysphagic for a variety of reasons including as a result of a stroke, neurodegenerative disorder or following radiotherapy or surgery affecting the oropharynx [2]. Patients may also have difficulty swallowing SODF in the absence of physiological difficulty often referred to as ‘pill aversion’[4]. Difficulty swallowing SODF can lead to non-adherence and/or medication administration errors [5, 6].

In the adult acute hospital patient setting, a recent study reported 15.4% of patients experienced difficulty with swallowing SODF [7]. This may necessitate the use of alternative routes of administration of a medicine or modification e.g. crushing a tablet [8]. In hospitals, the administration of medicines is usually performed by a nurse. However, other healthcare professionals (HCPs) may also be involved in the medicines management process e.g. doctors, pharmacists, dietitians and speech and language therapists (SALT) [9]. Organisational barriers such as lack of knowledge and resources can contribute to incorrect administration of medicines to patients [10]. SODF can be inappropriately modified to facilitate oral administration [6]. There is evidence that a need exists to guide, train and educate HCPs in medication management for patients with difficulty swallowing SODF [10–16].

A scoping review of knowledge and practice of HCPs’ reported competency gaps related to appropriateness of medicines modification, with pharmacists identified as the most appropriate HCPs to advise in this area [2]. Similarly another study also reported that, even in the absence of a formal multidisciplinary team (MDT), nurses tended to discuss patient medication requirements with pharmacists, doctors and where relevant SALT [17]. A qualitative evidence synthesis, described patient and HCPs knowledge, attitudes and beliefs regarding modification of SODF [18]. It highlighted that (i) individualised patient care (ii) the variability of swallowing difficulties, (iii) communication with both the patient and among the MDT, and (iv)evidence-based practice, were critical in providing optimal care [18].

Studies examining medicines administration to dysphagic patients often seek the opinion of nurses and less often the opinion of pharmacists or doctors, either in hospitals or aged-care facilities [2]. There are relatively few studies involving the broader HCP MDT [9] that are involved in medicines management for patients with difficulty swallowing medicines, specifically in an acute hospital setting.

To optimise care for these patients, it is important to undertake qualitative research examining the views of all HCPs involved in the care of this patient cohort, to inform the design of appropriate interventions to align with future behaviour change.

### Aim

To investigate HCPs’ views and identify resulting appropriate behaviour change strategies for HCPs to optimise the care of patients with difficulty swallowing SODF in an acute hospital setting.

## Method

### Study design

A qualitative interview study with semi-structured, in-person or online interviews with HCPs conducted between 3rd June and 12th July 2024. The study is reported in accordance with the Consolidated Criteria for Reporting Qualitative Studies (COREQ) (Online Resource 1).

### Study setting

Interviews were conducted at a large teaching hospital, admitting acute medical and surgical patients with an intensive care unit and a 24 h emergency department (ED), in the mid-west of Ireland.

### Inclusion criteria

Doctors, nurses, pharmacists, SALT and dietitians, involved in caring for patients (> 18 years) who have difficulty swallowing SODF, were eligible to participate. HCPs working exclusively with paediatric, psychiatric, day-care unit or outpatient cohorts were ineligible.

### Participant sampling and data collection

A combination of convenience and purposive sampling was used to recruit. Participants were selected because they belonged to one of the five HCP groups, with a view to including representation from a variety of grades within each group e.g. basic and senior grade, staff nurse and advanced nurse specialist etc. Recruitment posters were displayed in pharmacy reception and throughout the hospital inviting HCPs to volunteer for the study. Heads of Department were informed of the study and asked to publicise the study in their respective department. Eligible respondents were provided with a participant information sheet. All participants provided written informed consent. The semi-structured interviews were conducted using the Microsoft Teams function which facilitated recording of the interview and the generation of the initial transcript. A pilot interview was conducted but not included in the analysis.

A semi-structured interview guide (Online Resource 2) was used for each interview and underwent iterative change e.g. when new themes occurred. This was facilitated through review of the interview transcript by two reviewers after each interview. The interview guide questions were mapped to the Theoretical Domains Framework (TDF) (Online Resource 3). The TDF is an integrative framework of behavioural change theories with 14 core domains which facilitates identification of barriers and facilitators to implementation of best practice [19, 20]. It can inform behavioural change initiatives in a healthcare setting to improve patient outcome.

Interviews were conducted until there was representation across the sampling matrix, and in the final two interviews no new themes emerged.

### Data analysis

Interview transcripts were verified for accuracy by a research assistant and the PI independently. The final transcript was then approved, by the research team and the original recording was deleted. The transcripts were redacted to remove any identifying information. Pseudonyms using the profession of the participant (doctor, nurse, etc.) were assigned to study participants.

All redacted transcripts were read and reread to ensure familiarity and then uploaded to NVIVO software (Release1.7.2 (1560) May 2024) to facilitate data analysis. Transcripts were analysed independently by two researchers, one researcher coded transcripts directly into the TDF, with domains acting as pre-determined themes [20]. Independently, a second researcher conducted open coding generating non-hierarchical codes and categorising them into themes. These themes were then mapped into the TDF domains via a directed content analysis approach [20] All stages of coding were reviewed and agreed. Both coding approaches independently identified the same dominant TDF domains. These were subsequently mapped to the COM-B model and the Behavioural Change Wheel (BCW). The COM-B model cites Capability, Opportunity and Motivation as the factors which lead to specific behaviours, and influence behaviour change. This can then be utilised together with the BCW to inform what actions would be most beneficial to change behaviour and improve care for adult acute hospital patients with difficulty swallowing SODF [19, 21, 22].

### Ethics approval

The study received ethical approval from the Research Ethics Committee, University Hospital Limerick in March 2024 Ref 034/2024.

## Results

A total of thirteen HCPs were interviewed: 12 females, four doctors (consultant, registrar, senior house officer, intern), 3 nurses (two staff nurses, one Advanced Nurse Specialist), and a senior and basic grade dietitian, SALT and pharmacist. The average interview length was 22 min (range 12–32 min).

Eight of the fourteen TDF domains were identified as primary influencers of HCPs’ views of caring for acute hospital patients with difficulty swallowing SODF. The complete results are provided in Online Resource 4. The TDF domains, and conventional themes are presented in Table 1 [20].

**Table 1:** Dominant TDF Domains (definition and construct) and conventional themes

TDF Domain (D) Definition (C) Construct [20]	Conventional theme
*Memory attention and decision processes* D: The ability to retain information, focus selectively on aspects of the environment and choose between two or more alternatives C: Memory. Attention. Attention control (concentration). Decision making. Cognitive overload/tiredness	Importance of swallow assessment to inform decision making
	Alternative ways to manage oral medicines administration
	Decision aids and supports to identify patients
*Environmental context and resources* D: Any circumstance of a person’s situation or environment that discourages or encourages the development of skills and abilities, independence, social competence and adaptive behaviour C: Environmental stressors. Resources/material resources. Organisational culture/climate. Salient events/critical incidents. Person × environment interaction. Barriers and facilitators	Timely access to necessary resources
	Variable influence of environment on care
*Knowledge* D: An awareness of the existence of something C: Knowledge (including knowledge of condition/scientific rationale). Procedural knowledge. Knowledge of task environment	HCPs’ awareness of causes of difficulty swallowing SODF
	Knowledge gaps
*Social/professional role & identity* D: A coherent set of behaviours and displayed personal qualities of an individual in a social or work setting. C: Professional identity. Professional role. Social identity. Identity. Professional boundaries. Professional confidence. Group identity. Leadership. Organisational commitment	HCP roles & specific skill sets
	Importance of interdisciplinary process
*Goals* D: Mental representations of outcomes or end states that an individual wants to achieve C: Goals (distal/proximal). Goal priority. Goal/target setting. Goals (autonomous/controlled). Action planning. Implementation intention	Ensure patient can take their medications
*Beliefs about consequences* D: Acceptance of the truth, reality, or validity about outcomes of a behaviour in a given situation C: Beliefs. Outcome expectancies. Characteristics of outcome. Expectancies. Anticipated regret. Consequents	Consequences for the patient
	Consequences for the HCP
	Consequences for the organisation
*Behavioural Regulation* D: Anything aimed at managing or changing objectively observed or measured actions C: Self-monitoring. Breaking habit. Action planning	Best practice versus actual practice
	Steps to guide and improve HCP performance
*Intentions* D: A conscious decision to perform a behaviour or a resolve to act in a certain way C: Stability of intentions. Stages of change model. Transtheoretical model and stages of change	Unintentional non-adherence

### Memory, attention & decision processes

#### Importance of swallow assessment to inform decision making

 HCPs said that swallow assessment was crucial to decision making. The importance of immediate bedside assessment was highlighted, although the completion of the assessment in a timely manner was not always possible, with assessment in the ED being particularly challenging. HCPs confirmed that the absence of assessment led to decisions based on diagnosis, e.g. stroke patients are ‘nil by mouth’ until swallow assessment, with resultant missed doses. HCPs reported that bedside swallow assessment incorporating ‘the actual medication’ [SALT] would be beneficial.

*I think the most important thing would be for assessment of the swallowing of tablets, specifically to be a standard in every swallowing assessment.* [Dietitian]

#### Alternative ways to manage oral medicines administration

 All HCPs reported that alternative approaches to oral SODF administration were used. These included ‘crushing tablets [Nurse], ‘switching to a liquid’ [Doctor], ‘going on a patch’ [Pharmacist], ‘dispersible options’ [Doctor] intravenous or rectal preparation. Insertion of an enteral feeding tube and giving medicines in yogurt or other food was also reported.

*‘Like I suppose if somebody does have difficulties with swallowing tablets, first line nearly for all of them would be to crush them and to give them with a yogurt or something like that.’* [Dietitian]

Of note, HCPs reported that the swallowing aid Gloup™ was available on every ward but ‘they tend not to use it’ [SALT] with a preference for using yogurt or custard. HCPs reported concerns regarding appropriateness of modification methods including, (i) crushing sustained release preparations, (ii) whether formulations were interchangeable (iii) challenges with converting doses (iv) lack of familiarity with all the formulations available and (v) modification resulting in administration outside of product authorisation.

#### Decision aids & supports to identify patients

 HCPs reported many patients as easy to identify as having a swallowing difficulty e.g. those with Parkinson’s Disease or dementia. However, additional measures were required, when patients did not have a dysphagia diagnosis or diagnosis associated with swallowing difficulties. HCPs suggested inclusion of a question regarding swallowing ability as part of the admission documentation or Patient Medication Record. Placing a sign over the bed e.g. relating to the International Dysphagia Diet Standardisation Initiative level [23] or recording the difficulty on patient wristband were other suggested improvements as well as completion of a questionnaire on swallowing difficulty, to gather more detailed information.

*‘Even if there was something, in the kind of, the admission note that there’s a box that can be ticked, like, ‘has difficulties swallowing tablets.’* [Dietitian]

### Environmental context and resources

#### Timely access to resources

 Participants highlighted the need for access to ‘electronic databases’ [Doctor] on a desktop or phone app to guide decision making, e.g. to provide information on formulations available, alternative products or appropriate modifications.

*‘But it’d be nice to have some sort of repository or something that we could look at on call.’* [Doctor]

HCPs also noted the importance of adequate ‘staff numbers’ [Doctor] to allow effective multidisciplinary and interdisciplinary team working. HCPs reported that the ED lacked resources to facilitate access to the MDT ‘at the right time’ [SALT], for example in acute stroke where a patient might be without medicines due to being “nil by mouth”.

#### Variable influence of environment on care

Care for patients with difficulty swallowing SODF was reported to be superior in specialist wards such as the stroke unit due to specialist input, ‘higher staff ratio’ [Nurse], nurses being experienced in caring for this patient group and access to the MDT. In contrast, it was reported that ‘pitfalls’ [Doctor] included the general wards and ED where ‘things are done probably inappropriately’ [Nurse] due to not having specialist training and adequate MDT support. It was suggested that the introduction of cohort wards, grouping patients with difficulty swallowing oral medicines together, ensuring ‘you’ve trained staff’ [Nurse] and appropriate staff ratios and MDT access would enable better support.

*‘If you have cohort wards and people can get to the wards where you’ve trained staff with a focus, it’s, everything, it’s fabulous.* [Nurse]

### Knowledge

#### HCPs awareness of causes of difficulty swallowing SODF 

There was a high level of awareness of the patient cohorts associated with having difficulty swallowing SODF such as those with a diagnosis of Parkinson’s disease, stroke, dementia’ etc.

*‘Somebody with Parkinson’s who’s NPO, [nil per os] you know, that’s very difficult because you know they have to get their medication, and then you have the stroke patients.’* [Doctor]

HCPs were also aware of groups where ‘their swallow is technically fine’ [Pharmacist] but that may find it difficult to swallow medicines, with ‘anything big’ [Pharmacist] mentioned as a factor that affected patients’ ability to swallow medicines. All acknowledged that, outside of obvious cohorts such as stroke patients, it is difficult to know if any individual patient has a difficulty. HCPs highlighted red flags that should raise suspicion about the ability of the patient to swallow such as asking about the size of a medicine, coughing after oral intake or being diagnosed with aspiration pneumonia.

#### Knowledge gaps 

HCPs reported knowledge gaps and educational needs.

*‘So, I think there is there’s a gap in in knowledge of doctors and of the route of, administration of these drugs and which drugs to be given.’* [Doctor]

Inclusion of swallowing difficulties in the undergraduate curriculum for HCPs was suggested, in addition to better access to postgraduate training.

### Social/professional role & identity

#### HCP roles and specific skill sets

 Participants reported that care for this patient cohort was improved when specific HCPs were available for advice.

*‘Maybe they need another Speech & Language review, or maybe they need to liaise with pharmacy if there are different versions [of medicine] that they can have.’* [Dietitian]

HCPs reported the importance of pharmacist involvement and a reliance on pharmacist advice regarding available formulations and permitted modifications for example Doctor stated ‘if in doubt always ask pharmacy’. However, they felt that this advice was not readily available ‘out-of-hours’ [Nurse] when the pharmacist was not on duty in the evening, at night and on weekends. Nurses experienced in care for this cohort and the role of the SALT was also considered crucial.

#### Importance of interdisciplinary process

 HCPs felt that involvement of the MDT and interdisciplinary working was essential in caring for this patient cohort.

*‘We get the MDT members to just come in and just give us their opinion and see what is the best thing for them (the patient).’* [Doctor]

Value was placed on having ‘somebody to ask’ [Dietitian], the ability to consult with ‘MDT members’ [Doctor] to be able to make a referral and get a different perspective.

### Goals

#### Ensure patient can take their medicines

 HCPs considered that being able to take essential medications in a formulation that facilitates safe administration, was critical. This was reported as essential where anticoagulants or antiepileptics are indicated and in Parkinson’s Disease.

*‘I think we have to be conscientious on how, which medications are prescribed for the patient [with difficulty swallowing] and in what formulation, and deprescribing is a big part of our job.’* [Doctor]

### Beliefs about consequences

#### Consequences for the patient

 HCPs emphasised the prevalence of the oral route for medicines administration and thus the risks, including aspiration or choking, had to be fully considered. There was acknowledgement of the prognostic difference between those with an acute versus chronic swallowing difficulty.

*‘Especially when it’s a new issue [swallowing difficulty] for a patient because it can often be debilitating for them not to be able to eat or drink something orally, let alone tablets. I mean, if ‘You have a patient who’s had ongoing swallowing, that’s a different scenario because they will have kind of adjusted ways around to be able to take their tablets’* [Doctor]

HCPs reported that an overly cautious approach such as having all stroke patients ‘nil by mouth’ until swallow assessment, potentially leads to patients ‘floating in limbo [Pharmacist] missing doses when the route of administration has not been changed, and the assessment does not take place in a timely manner.

#### Consequences for the HCP

 All agreed that there was a greater workload attached to caring for this patient group than caring for those without the difficulty.

*‘I suppose it means extra work in terms of just trying to overcome and taking it [the difficulty swallowing SODF] into account.’* [Nurse]

HCPs reported stressful feelings when faced with dilemmas in certain situations such as how to administer a SODF or determining whether an alternative formulation existed.

#### Consequences for the organisation

 HCPs believed that developing a difficulty with swallowing SODF could increase length of stay in hospital for example if the patient developed an aspiration pneumonia because of medicines ‘going down the wrong way’ [SALT].

*‘And I just think when we’re so focused on, kind of, shorter amount of bed stays and things like that—it [difficulty swallowing SODF] could just have a huge impact’* [SALT]

Increasing length of stay was recognised as being costly for the organisation and potentially wasted limited resources.

### Behavioural regulation

#### Best practice versus actual practice

 HCPs admitted that it was important to ‘align to the best approach’ [Doctor] but that best practice was not always possible or feasible.

*‘It doesn’t necessarily need to be the best as well, it’s, it’s what’s feasible within our environment that often works.’* [Doctor]

Audit was considered an important measure of best practice. HCPs spoke about modifications being made to medicines to facilitate administration with the potential for the modification to be inappropriate for example, crushing of slow-release tablets [Dietitian] or ‘enteric coated drugs [Doctor]. HCPs also raised how modifications are most often outside of the medicine’s authorisation, which may raise legal sequelae.

#### Steps to guide and improve HCP performance

 Participants proposed several actions that could be taken to enhance care for these patients including the provision of guidelines and policies for HCPs, having ‘a clear pathway’ [Dietitian], standardising the approach to medicines management in patients with swallowing difficulty and having ‘a medication administration plan documented’ [SALT]. HCPs were concerned about there being ‘no standard practice’ [SALT] in caring for those with swallowing difficulty and wanted greater integration and standardisation of systems of care.

*“So, you know, even if it was like a pathway in the nursing guidelines.* [Pharmacist]

### Intentions

#### Unintentional non-adherence

 Participants reported that adherence to a specific treatment regimen was difficult when a patient had difficulty swallowing SODF. There could be a delay in medicines administration, doses could be missed, medicines could be refused due to difficulty with swallowing and errors could be made in the modification of medicines.

*‘And then they’ve missed five, you know, four or five days of medications and then that becomes an issue.’* [Doctor]

### Application of the COM-B and the BCW

The principles of the COM-B model and the BCW were applied to the TDF findings (Table 2). Interventions targeting Capability, Opportunity and Motivation were all identified as being potentially successful, with low-cost interventions such as recording the patient or carer response to a question on swallowing at admission and some longer-term approaches such as increasing the number of HCPs.

**Table 2:** Suggested strategies, for HCPs, identified by applying the TDF and COM-B to the study findings, [20, 21]

Target behaviour	COM-B factor	TDF domain	Strategy example
Support Medication adherence	Capability	Intentions Knowledge	Refer patient to pharmacist for medicines review Involve MDT in patient care Access decision support tools for example to facilitate formulation change
Timely Identification of patients with swallowing difficulty	Capability	Memory, attention and decision processes	Introduce self-assessment questionnaire for patient or carer e.g. SWAMECO, PILL5 Perform swallow assessment Incorporate tick box answer to question on ability to swallow medicines
Removal of barriers to timely swallow assessment	Capability	Memory, attention and decision processes	Advocate for adequate numbers of SALTs Promote development of bed-side swallow assessment by non-SALTs
Consider patient ward placement	Opportunity	Environmental context & resources	Promote appropriate cohorting of patients Lobby for enhanced HCP resources in ED
Avoid erroneous modification of SODFs	Capability Motivation	Knowledge Memory, attention and decision processes	Educate HCPs Promote appropriate cohorting of patients Lobby for enhanced HCP to bed ratio Introduce individual medicines management plan

## Discussion

### Main findings

This study reported HCPs views of caring for patients with difficulty swallowing SODF, using behavioural change theory to inform improvements in care. Key findings included recognition of challenges in identifying patients in the cohort, assessing ability to swallow medicines in real time and having access to resources to guide decision making. HCPs raised concerns regarding consequences for patients, HCPs and the organisation. Participants suggested changes including MDT involvement, education and training Study findings will inform appropriate next steps in the development of care pathways. The COM B highlighted intervention strategies that may help to modify HCPs behaviour when caring for these patients. The robust use of behaviour change theory in addition to the novelty of the findings provide real-world evidence to inform the development of interventions to address the challenge of swallowing difficulties in medication management.

### Interpretation

The TDF domain of Memory, Attention and Decision Processes was dominant and revealed significant challenges when caring for these patients. Environmental context and Resources was also a dominant domain and highlighted the difference between care for this group on the stroke unit versus a general medical ward. Knowledge deficits were acknowledged and the challenges of effective interdisciplinary team working and access to HCPs with appropriate skills, contributed to a high ranking for the TDF domains of Knowledge and Social/Professional Role and Identity respectively.

The challenges for HCPs when caring for patients with difficulty swallowing SODF in an acute hospital, were consistent with those seen in other studies [18]. A qualitative focus group study of HCPs described the problems experienced when caring for these patients. [9]. Challenges raised in that study echoed some of the challenges identified here, e.g. challenges with identification of these patients, choosing suitable formulations and the absence of decision supports for HCPs.

COM-B strategies to help HCPs to identify patients, included addition of an admission question on ability to swallow medication, an approach that is also recommended in guidelines [24, 25]. HCPs also suggested a carer or patient completed questionnaire to gather data to assist them in management. The use of a validated patient-completed questionnaire in an acute hospital has provided valuable information and helped to identify this patient cohort [5]. Here HCPs suggested the Pill-5 questionnaire, with evidence in the literature to support effectiveness of this approach [5, 26].

HCPs proposed that cohorting on wards to maximise availability of staff experienced in managing swallowing difficulty issues, facilitated efficient MDT working and improve management overall. Cohorting is implemented primarily in infection prevention and control [27, 28]. In this case, patients with difficulty swallowing medicines have a higher morbidity level [29] and thus admission to a unit area best equipped to treat their primary diagnosis may be more appropriate.

The ED was highlighted as being an area of the admission pathway where patient harm could occur due to patients missing doses of medication. HCPs advocated that care for these patients required priority swallow assessment with MDT input from doctors, pharmacists and nurses to ensure patients had access to essential medications. Enhanced HCP resources are required for dysphagia management in the ED, and these resources may also help in the identification of the most appropriate ward for onward care [30]. The relevance of the acuity and chronicity of the swallowing difficulty was also recognised e.g. in the management of an acute stroke, the swallowing difficulty may be of recent onset and depending on recovery and level of disability, may improve [31]. In contrast, a patient with Parkinson’s disease may experience another reason for acute admission, on a background of a chronic potentially fluctuating swallow [32].

The importance of swallow assessment was highlighted. The gold standard is for swallow assessment by SALT [33]. Bedside swallow assessment by non-SALT grades is also used but is more likely to produce inaccurate results [33]. This study’s findings indicate that swallow assessment should be completed as soon as possible to reduce patient risk [25].

Education, training and resources for HCPs was desired, especially on medicines modification and formulation choice. Previous studies have highlighted that nurses desired increased education and resources on medicines modifications [34] and that there was a need for prescribers to be educated on the appropriateness and modification of SODF [35]. However, information resources alone are not always sufficient if adequate staff training is not provided [13]. HCPs raised concerns regarding erroneous modification of oral medicines with potential negative consequence, a risk that is well documented [10–16]. Warning labels on medicines advising not to crush have been successful at reducing inappropriate modification [16]. Organisational level commitment to safe practice when administering medicines to patients with difficulty swallowing SODF, should not be underestimated [10].

Identification of difficulty swallowing SODF allows issues such as non-adherence due to the swallowing difficulty to be addressed before it adversely impacts patient care and outcomes [25]. Identification of the difficulty also decreases patient risk of choking or oesophageal damage if the oral dosage form lodges in the throat and is corrosive [25]. HCPs reported a reliance on pharmacists’ advice regarding available formulations and permitted modifications. Pharmacist-led services are successful in improving adherence [36]. Decision supports, particularly if the pharmacist is not accessible, were highlighted as necessary for HCPs [17]. These could include the use of electronic resources [37, 38]. The importance of pharmacist involvement has also been highlighted in relation to medicines management in this patient cohort [8, 39].

The value of interprofessional working among HCPs was a common theme across all interviews, and has been previously highlighted [9, 14, 15, 35]. Lau et al. [40] recommended that, until novel dosage forms are available, effective interdisciplinary team working is crucial to reducing risk where medicines are modified to facilitate administration. Kelly et al. also reported this finding among interviewed nursing staff in long-term care and acute care facilities [34]. It is important therefore for those planning services to consider the value of the MDT approach and ensure that interdisciplinary team working is prioritised in recruitment and retention policies for hospitals.

### Strengths and limitations

This study had several key strengths. Participant representation was obtained from all relevant professions and career stages in the acute hospital setting. The mapping of the interview topic guide questions to the TDF allowed for consistency across the core topics, with a recognised behavioural change framework to identify future interventions, of HCPs, to optimise patient care. Data was collected until no new themes emerged [41].The provision of illustrative quotes from participants in the reporting of results enhanced confirmability. Finally, many of the participants were experienced HCPs who had worked in many acute hospitals both in Ireland and abroad, however the study recruited HCPs from a single acute hospital which limits the context and potentially its transferability. The research team were predominately pharmacists and while we acknowledge that qualitative research is inherently interpretive, we engaged in reflexivity to confront our own biases and preconceptions and how our views may have influenced the data and its analysis. To mitigate this peer debriefing was conducted to challenge any assumptions and broaden our interpretative scope. Finally, future work should include the patient voice to expand understanding of lived experience.

## Conclusion

HCPs recommend the timely identification of patients with difficulty swallowing SODF to ensure access to appropriate medication formulations. MDT involvement was considered to offer substantial advantages in terms of patient access to appropriate medications and formulations. Decision support tools were valued, as was education for HCPs. HCPs warned that unintentional non-adherence and inappropriate modification of SODF could result from failure to adequately recognise or manage a patient with difficulty swallowing SODF. The COM-B identified interventions for HCPs, to improved outcomes and may be useful to other institutions where similar difficulties arise.

## Supplementary Information

Below is the link to the electronic supplementary material.

Supplementary file1 (DOCX 53 KB)

Supplementary file2 (PDF 203 KB)

Supplementary file3 (PDF 203 KB)

Supplementary file4 (PDF 173 KB)

## Data Availability

The ethics application in this study neither sought nor received permission to share participants interviews publicly.
