# Supplementary material for: A qualitative study exploring the views of healthcare professionals regarding patients who have difficulty swallowing medicines
Source: Int J Clin Pharm. 2026 Apr 1;48(4):1500–9. doi: 10.1007/s11096-026-02129-9 (PMC13368901; doi:10.1007/s11096-026-02129-9)
Supplement: Supplementary file 1 — Supplementary file1 (DOCX 53 KB) [file 11096_2026_2129_MOESM1_ESM.docx]

A qualitative interview study of the views of healthcare professionals regarding patients who have difficulty swallowing medicines.

**A Harnett 1,2; C Murphy 1; L J Sahm 1,3; S Byrne 1; D Lyons 2; M O’Driscoll 1.**

**1: Pharmaceutical Care Research Group, School of Pharmacy, University College Cork, Cork, Ireland**

**2: University Hospital Limerick, Dooradoyle, Limerick, Ireland**

**3: Pharmacy Department, Mercy University Hospital, Grenville Place, Cork, Ireland**

International Journal Of Clinical Pharmacy

Corresponding author Anne Harnett anne.harnett1@hse.ie

**Online Resource 1 Consolidated Criteria for Reporting Qualitative Studies (COREQ) 32-item checklist** (38)**.**

| **Domain 1: research team and reflexivity** | | |
| --- | --- | --- |
| **Personal characteristics** | | |
| 1. Interviewer/facilitator | Which author(s) conducted the interview or focus group? | The lead author (AH) and the research assistant (CM). |
| 2. Credentials | What were the researcher’s credentials? (e.g. PhD, MD) | AH is a PhD candidate in year 5 of a six-year PhD. She has a Post Graduate Master’s in Clinical Pharmacy and a Master’s in Medical Education. CM is an undergraduate pharmacy student in year 4 of a 5-year MPharm programme. |
| 3. Occupation | What was their occupation at the time of the study? | AH is a pharmacist Executive Manager of a University Hospital and a PhD student. CM is a undergraduate pharmacy student. |
| 4. Gender | Was the researcher male or female? | AH is female. CM is male. |
| 5. Experience and training | What experience or training did the researcher have? | AH undertook training in qualitative research methodologies and has previous experience of this methodology |
| **Relationship with participants** | | |
| 6. Relationship established | Was a relationship established prior to study commencement? | No relationship was established between the researchers and participants prior to interviews. |
| 7. Participant knowledge of the interviewer | What did the participants know about the researcher? (e.g. personal goals, reasons for doing the research) | Participants knew the researcher’s role within the hospital and the purpose of the research. |
| 8. Interviewer characteristics | What characteristics were reported about the interviewer/facilitator? (e.g. bias, assumptions, reasons and interests in the research topic) | Affiliations & declaration of interests |
| **Domain 2: study design** | | |
| **Theoretical framework** | | |
| 9. Methodological orientation and theory | What methodological orientation was stated to underpin the study? (e.g. grounded theory, discourse analysis, ethnography, phenomenology, content analysis) | Phenomenology |
| **Participant selection** | | |
| 10. Sampling | How were participants selected? (e.g. purposive, convenience, consecutive, snowball) | A combination of convenience and purposive sampling was used to recruit participants. Participants were selected because they belonged to one of the five HCP groups of interest (doctor, nurse, speech and language therapist (SALT), dietitian, pharmacist) with a view to having a variety of grades within each group e.g. basic and senior grade, staff nurse and advanced nurse specialist, consultant, registrar, intern etc. |
| 11. Method of approach | How were participants approached? (e.g. face to face, telephone, mail, e-mail) | Posters were displayed in the pharmacy reception area and throughout the hospital inviting HCPs to volunteer for the study with contact details for the researchers. Heads of Department were informed of the study by email and asked to publicise the study in their respective departments. Participants contacted the researchers by phone, email or in person at the hospital indicating their willingness to participate in the study. Eligible respondents were provided with a participant information sheet and if they agreed to participate, they signed an informed consent form. |
| 12. Sample size | How many participants were in the study? | A total of thirteen HCPs were interviewed: 12 females, four doctors (consultant, registrar, senior house officer, intern), 3 nurses (two staff nurses, one Advanced Nurse Specialist), and a senior and basic grade dietitian, SALT and pharmacist. |
| 13. Non-participation | How many people refused to participate or dropped out? Reasons? | No participants refused to participate or dropped out of the study. |
| **Setting** | | |
| 14. Setting of data collection | Where was the data collected? (e.g. home, clinic, workplace) | Semi-structured interviews with staff took place in the hospital. |
| 15. Presence of non-participants | Was anyone else present besides the participants and researchers? | Only researchers were present with participant. |
| 16. Description of sample | What are the important characteristics of the sample? (e.g. demographic data, date) | The HCP participant’s profession and grade was included in the study. For each participant the following was provided: their years of experience and gender. |
| **Data collection** | | |
| 17. Interview guide | Were questions, prompts, guides provided by the authors? Was it pilot tested? | An iterative semi-structured interview guide was developed. This guide was pilot tested with one participant prior to commencement of the data collection process. |
| 18. Repeat interviews | Were repeat interviews carried out? If yes, how many? | No repeat interviews were carried out. |
| 19. Audio/visual recording | Did the research use audio or visual recording to collect the data? | Semi-structured interviews were audio or video recorded. |
| 20. Field notes | Were field notes made during and/or after the interview or focus group? | No |
| 21. Duration | What was the duration of the interviews or focus group? | The average interview length was 22 minutes (range 12-32 minutes). |
| 22. Data saturation | Was data saturation discussed? | A maximum variation sampling matrix was used to ensure adequate representation from HCP groups. In addition, interviews were conducted until no new themes emerged. |
| 23. Transcripts returned | Were transcripts returned to participants for comment and/or correction? | Transcripts were not returned to participants for comment or correction. |
| **Domain 3: analysis and findings** | | |
| **Data analysis** | | |
| 24. Number of data coders | How many data coders coded the data? | One researcher coded transcripts directly into the TDF.  Independently a second researcher conducted open coding generating non-hierarchical codes and categorising them into themes. These themes were then mapped into the TDF domains in a directed content analysis approach.  All stages of coding were reviewed and agreed.  Both coding approaches independently identified the same dominant TDF domains. |
| 25. Description of the coding tree | Did authors provide a description of the coding tree? | The description of coding is provided in methods section on analysis. |
| 26. Derivation of themes | Were themes identified in advance or derived from the data? | One researcher coded transcripts directly into TDF.  Independently, a second researcher conducted open coding generating non-hierarchical themes. These themes were then mapped to the TDF using a directed content analysis approach. Both coding approaches independently identified the same dominant TDF domains. |
| 27. Software | What software, if applicable, was used to manage the data? | NVIVO software (Release1.7.2 (1560) May 2024) |
| 28. Participant checking | Did participants provide feedback on the findings? | No |
| **Reporting** | | |
| 29. Quotations presented | Were participant quotations presented to illustrate the themes/findings? Was each quotation identified? (e.g. participant number) | Quotations are presented with participant code e.g. HCP1 used to identify the participant. |
| 30. Data and findings consistent | Was there consistency between the data presented and the findings? | Yes, we reported the study findings as they related to the original interviews. |
| 31. Clarity of major themes | Were major themes clearly presented in the findings? | Yes, major themes are clearly presented in the results section and discussed in the text under Table 2. |
| 32. Clarity of minor themes | Is there a description of diverse cases or discussion of minor themes? | There were no minor themes. |
