# Supplementary material for: A qualitative study exploring the views of healthcare professionals regarding patients who have difficulty swallowing medicines
Source: Int J Clin Pharm. 2026 Apr 1;48(4):1500–9. doi: 10.1007/s11096-026-02129-9 (PMC13368901; doi:10.1007/s11096-026-02129-9)
Supplement: Supplementary file 2 — Supplementary file2 (PDF 203 KB) [file 11096_2026_2129_MOESM2_ESM.pdf]

A qualitative interview study of the views of healthcare professionals regarding patients who have difficulty swallowing medicines.

A Harnett 1,2; C Murphy 1; L J Sahm 1,3; S Byrne 1; D Lyons 2; M O'Driscoll 1.

1: Pharmaceutical Care Research Group, School of Pharmacy, University College Cork, Cork, Ireland

2: University Hospital Limerick, Dooradoyle, Limerick, Ireland

3: Pharmacy Department, Mercy University Hospital, Grenville Place, Cork, Ireland

International Journal Of Clinical Pharmacy

Corresponding author Anne Harnett anne.harnett1@hse.ie

Online Resource 3 Mapping the topic guide to the TDF.

Interview Guide mapped to TDF – HCP interviews regarding patients with difficulty swallowing SODF.

<u>Interview Guide</u>

1)

Question Number	Question	TDF domains
**Area 1**	**Experience with patients with difficulty swallowing solid oral dose forms.**	
1	Could you tell me briefly about your experience with patients who have difficulty swallowing SODF, for example tablets and capsules?<br><u>Prompt:</u> What are the challenges that you encounter when caring for patients with difficulty swallowing solid oral dose forms such as tablets and capsules?<br>How would you describe the effect those challenges have on your professional practice?	1,2,3,<br>6,7,12<br>3, 4, 5,6,
2	How do you identify patients with difficulty swallowing solid oral dose forms?<br>Prompt: Identification challenges	2, 4, 11
3	What are your views on medication adherence in these patients?	6, 8, 12
4	What is your experience of the differences in medication administration for patients with swallowing difficulty (versus those without)?	8, 10,11
**Area 2**	**Managing patients with difficulty swallowing solid oral dose forms.**	
1	Could you tell me about how you help the patient to manage this difficulty with swallowing SODF?	1,2,3,4,5,9,10,12,13,14
	How do you feel when caring for patients who have difficulty swallowing SODF?<br><u>Prompt:</u> stress re decision making, concerns – legal, ethical, therapeutic, pharmaceutical	3, 4, 5, 6, 7, 8, 11, 12,13,
**Area 3**	**Current standard of management of patients with difficulty swallowing SODF.**	

1	What are your views on your knowledge and skill to confidently care for a patient with difficulty swallowing SODF?<br>Prompt: Do you feel that you have been provided with adequate training, equipment, workspace, time, access to guidelines, access to export advice to confidently & safely care for those patients?	1,2,3,4,9,13,14
**Area 4**	**Attitudes, values, and preference about how the difficulty of swallowing SODF should be managed.**	
1	If you could re-design the approach that is taken to help patients manage their difficulty with swallowing SODF, what would be the most important elements of the approaches taken?<br>Prompt: Other healthcare professionals	1,2,3,4,5
2	Would you recommend any suggestions for adaptations to work practice in relation to caring for patients with swallowing difficulties?<br>Prompt: can you explain why you would or would not recommend such changes?	5,9,12,13
3	Is there anything else that you would like to comment on about caring for patients with difficulty swallowing solid oral dose forms?	
