# Supplementary material for: A qualitative study exploring the views of healthcare professionals regarding patients who have difficulty swallowing medicines
Source: Int J Clin Pharm. 2026 Apr 1;48(4):1500–9. doi: 10.1007/s11096-026-02129-9 (PMC13368901; doi:10.1007/s11096-026-02129-9)
Supplement: Supplementary file 4 — Supplementary file4 (PDF 173 KB) [file 11096_2026_2129_MOESM4_ESM.pdf]

# A qualitative interview study of the views of healthcare professionals regarding patients who have difficulty swallowing medicines.

**A Harnett 1,2; C Murphy 1; L J Sahm 1,3; S Byrne 1; D Lyons 2; M O'Driscoll 1.**

**1: Pharmaceutical Care Research Group, School of Pharmacy, University College Cork, Cork, Ireland**

**2: University Hospital Limerick, Dooradoyle, Limerick, Ireland**

**3: Pharmacy Department, Mercy University Hospital, Grenville Place, Cork, Ireland**

International Journal Of Clinical Pharmacy

Corresponding author Anne Harnett anne.harnett1@hse.ie

**Online Resource 4 Frequency of coded domains from TDF**

The frequency of coding for each of the 14 TDF domains is provided in the table below. The first 8 domains were considered to be the predominant domains that influenced HCPs HCP perceptions and experiences of caring for patients with difficulty swallowing SODF.

| Domain | Number of transcripts where domain was coded | Frequency of coding in transcripts |
|---|---|---|
| Memory, attention and decision processes | 13 | 444 |
| Environmental context and resources | 13 | 279 |
| Knowledge | 13 | 202 |
| Social/professional role and identity | 13 | 161 |
| Goals | 12 | 129 |
| Beliefs about consequences | 13 | 121 |
| Behavioural regulation | 12 | 101 |
| Intentions | 13 | 75 |
| Emotion | 10 | 44 |
| Skills | 9 | 35 |
| Social influences | 4 | 16 |
| Beliefs about capabilities | 6 | 11 |
| Optimism | 4 | 7 |
| Reinforcement | 2 | 2 |
